# Syntheses of hydroxyapatite from natural sources

**DOI:** 10.1016/j.heliyon.2019.e01588

**Published:** 2019-05-08

**Authors:** N.A.S. Mohd Pu'ad, P. Koshy, H.Z. Abdullah, M.I. Idris, T.C. Lee

**Affiliations:** a Department of Production and Operation Management, Faculty of Technology Management and Business, Universiti Tun Hussein Onn Malaysia, 86400 Batu Pahat, Johor, Malaysia; b School of Materials Science and Engineering, UNSW, Sydney, NSW 2052, Australia; c Department of Materials Engineering and Design, Faculty of Mechanical and Manufacturing Engineering, Universiti Tun Hussein Onn Malaysia, 86400 Batu Pahat, Johor, Malaysia

**Keywords:** Biomedical engineering, Natural product chemistry, Biological sciences, Biotechnology, Health sciences

## Abstract

Waste materials from natural sources are important resources for extraction and recovery of valuable compounds. Transformation of these waste materials into valuable materials requires specific techniques and approaches. Hydroxyapatite (HAp) is a biomaterial that can be extracted from natural wastes. HAp has been widely used in biomedical applications owing to its excellent bioactivity, high biocompatibility, and excellent osteoconduction characteristics. Thus, HAp is gaining prominence for applications as orthopaedic implants and dental materials. This review summarizes some of the recent methods for extraction of HAp from natural sources including mammalian, aquatic or marine sources, shell sources, plants and algae, and from mineral sources. The extraction methods used to obtain hydroxyapatite are also described. The effect of extraction process and natural waste source on the critical properties of the HAp such as Ca/P ratio, crystallinity and phase assemblage, particle sizes, and morphology are discussed herein.

## 1 Introduction

Biomaterials have been gaining increasing importance owing to their applicability to ageing populations and treatment of diseases. Research on developing new biomaterials or manipulating the structure and composition of existing biomaterials has been intensively focussed in order to enhance the properties of biomedical devices. In general, biomaterials are commonly used as implants, tissues, and organ transplants and in drug delivery systems [1]. The biomaterials act to restore, repair, or replace the damaged tissue by integrating with the problematic part of the body in order to increase the life expectancy [2]. The diverse mechanical, physical, chemical, and structural properties of biomaterials allow them to be used in various applications depending on the biocompatibility and characteristics.

Ceramics are a class of biomaterials used in biomedical devices. Ceramics are widely used as implant materials owing to their ability to be fabricated into a variety of shapes, along with their high compressive strength, variable porosity, and bioactive properties in the body [3]. The high similarity in the chemical composition of some ceramics such as calcium phosphate with human bone minerals makes them suitable for use as orthopaedic implants (human skeleton, bones, and joints), and dental materials [4]. These materials show excellent bioactivity, high biocompatibility, and excellent osteoconduction characteristics [3, 5].

Hydroxyapatite, HAp (Ca_10_(PO_4_)_6_(OH)_2_), is thermodynamically stable in its crystalline state in body fluid and has a very similar composition to bone mineral [6]. HAp can integrate with bone without causing any local or systemic toxicity, inflammation or foreign body response [7]. For these reasons, HAp has been widely used for biomedical applications particularly in orthopaedic, odontology, and as the coating material for metallic implants [8]. Consequently, methods for synthesising HAp with customisable characteristics have been extensively studied. Although many synthesis methods have been developed, the preparation of HAp with specific characteristics still remains challenging because of the possibility of formation of toxic intermediary products during the synthesis of HAp [6]. Therefore, studies on new parameters of synthesising HAp are still ongoing.

HAp can be synthesised chemically or extracted from natural sources. Prior research has reported on the various methods for synthesising synthetic and natural HAp. The review by Sadat-Shojai *et al*. concluded that synthetic HAp can be fabricated through various methods including dry methods (solid-state and mechanochemical), wet methods (chemical precipitation, hydrolysis, sol-gel, hydrothermal, emulsion, and sonochemical), and high temperature processes (combustion and pyrolysis) [6]. Compared to synthetic HAp, natural HAp is non-stoichiometric since it contains trace elements such as Na^+^, Zn^2+^, Mg^2+^, K^+^, Si^2+^, Ba^2+^, F^−^, and CO_3_^2-^ which make it similar to the chemical composition of human bone [9, 10].

This review provides an in-depth review of a series of methods for extracting HAp from natural sources. Studies and work done for the past 20 years in extracting HAp from mammalian sources, aquatic and marine sources, shell sources, plants and algae, and mineral source are discussed thoroughly. Additionally, this review also investigates the effect of sources and processing methods on the properties such as Ca/P ratio, particle size and morphology, and crystallinity and phase assemblage of natural HAp since the performance of the synthesised HAp is influenced by its crystallinity, size, and morphology [11]. This review thus provides a benchmark for further advanced studies on utilising natural HAp.

## 2 Main text

### 2.1 Natural hydroxyapatite

**Fig. 1:**
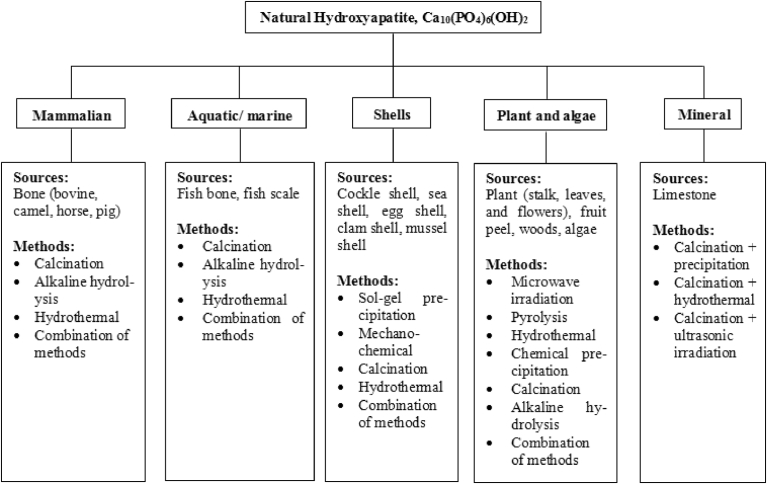
Summary of processes for synthesising natural HAp.

Natural hydroxyapatite is usually extracted from biological sources or wastes such as mammalian bone (e.g. bovine, camel, and horse), marine or aquatic sources (e.g. fish bone and fish scale), shell sources (e.g. cockle, clam, eggshell, and seashell), and plants and algae and also from mineral sources (e.g. limestone). Fig. 1 shows the sources and examples of techniques used for synthesising natural HAp. Stoichiometric HAp is basically composed of calcium and phosphorus with molar ratio of Ca/P equal to 1.67. This ratio has been proven to be the most effective in promoting bone regeneration [9]. Natural HAp is non-stoichiometric and is either deficient in calcium or phosphorus [12]. Calcium positions are the most common vacancy in HAp where cations such as Na^+^, Mg^2+^, and Al^3+^ are substituted in the calcium positions, while carbonate ions can substitute for either phosphate or hydroxyl ions while fluoride ions substitute for hydroxyl ions [12].

The presence of trace elements in some natural HAp mimics the apatite produced from human bone [13]. The trace elements are essential in the regeneration of the bone and accelerates the process of bone formation [9]. Research by Balamurugan *et al.* proved that 3–5 mol% silicon added to synthetic hydroxyapatite (Si-HAp) increased cell growth density which enhanced osteoblast growth [14]. Research by Capuccini *et al.* showed that the presence of 1–10% of strontium ions in synthetic HAp enhanced osteoblast activity and differentiation and also inhibited osteoclast proliferation and production [15]. Carbonate-substituted hydroxyapatite was also proven to enhance osteogenesis by enhancing bioresorption [16].

The usage of HAp extracted from natural sources can be considered to be an environmentally friendly, sustainable, and economical process to fabricate these materials since these materials are available in large quantities. This can result in positive contributions to the economy, environment, and to general health.

### 2.2 Extraction of hydroxyapatite (HAp) from natural sources

#### 2.2.1 Mammalian sources

Among mammalian sources, the extraction of HAp from bovine bone was frequently reported in literature compared to other sources such as camel, horse and, porcine. The cortical part of the femoral bone is usually used because they are morphologically and structurally similar to human bone [17]. Reviewing the literature shows that the properties of the extracted HAp, such as the Ca/P ratio, size, shapes and crystalline phases of Ca-P have been discussed. These properties differ with the applied extraction methods and thus the parameters such as calcination temperatures and pH.

Generally, most literature have reported that pretreatment of the bone is usually done before proceeding with the extraction method. The pretreatment involves washing and removing the dirt, fats, protein, and other components such as bone marrows and soft tissues. Some literature reported the usage of boiling water to remove organic components from the bone by boiling for times of 8 h or more [18, 19]. A combination of boiling and washing with solvents such as acetone and chloroform have been employed for the pretreatment of bone [20, 21]. Another pretreatment method that has been widely used is washing the bone alternatively with surfactant and alkali solutions to remove the soft tissues and decellularise it [22]. The bone was also cut into smaller pieces before or after removing the organic constituents. Most majority of literature reported that the bone was cut first into smaller pieces before boiling or treated with the solvent to remove the unwanted components such as bone marrow located inside the bone [19, 20, 21, 22, 23].

In this review, most of the methods for extraction of HAp from mammalian bones used the calcination method which is either the sole process or a combination of calcination with other methods. The calcination process involves heating the bone in a furnace at different temperatures of up to 1400 °C in order to completely remove the organic matter and kill the pathogens which may be present [9].

Barakat *et al.* employed the alkaline hydrothermal hydrolysis treatment to extract HAp from bovine bone [20]. The extracted HAp was heated to 250 °C for 5 h resulting in the formation of nanoflake HAp with Ca/P ratio of 1.86. They also reported that nanoflake HAp with Ca/P ratio of 1.56 could be produced using subcritical water process [20]. Meanwhile, Hosseinzadeh *et al.* extracted HAp using thermal decomposition method where samples were heated at two different temperatures (750 °C and 850 °C) for 6 h [21] leading to the formation of HAp with two particle sizes, namely fine (<420 μm) and coarse (420–500 μm) at 750 °C and 850 °C, respectively [21]. From XRD results, the bone powder heated at 750 °C exhibited pure HAp while at 850 °C, a combination of β-tricalcium phosphate (β-TCP) and HAp was observed. The Ca/P ratio of the sample heated at 850 °C reduced to 1.5 owing to the presence of β-TCP. Ayatollahi *et al.* [18] also employed heat treatment to produce HAp from bovine bone with calcination done at 900 °C for 2 h. The calcined bone was milled using high energy planetary ball mill to reduce the size to 30 nm. XRD results showed that pure HAp phase was obtained at 900 °C. Sun *et al.* also employed calcination method to extract HAp from bovine bone by heating at 750 °C for 2 h. This process resulted in the formation of irregularly shaped HAp with size of 20–100 μm and Ca/P ratio >1.67 [22]. The Ca/P ratio was higher than 1.67 owing to the exchange of ions such as carbonate in the HAp structure [22]. XRD patterns showed that HAp and β-TCP were formed during the calcination. Previous research showed that HAp decomposes to β-TCP at temperatures >1000 °C, but research by Hosseinzadeh *et al.* [21] and Sun *et al.* [22] showed that this process can occur at 850 °C [21] and 750 °C [22] leading to formation of β-TCP. Thus Sun *et al.* concluded that the presence of β-TCP was not formed from the decomposition of HAp and thus may have originated from the bovine bone [22].

Sun *et al.* used alkaline heat treatment to extract HAp from bovine bone [22] where the sample was treated with 20% sodium hydroxide and heated at 80 °C for 10 h followed by washing and freeze drying. This process resulted in low-crystalline irregularly-shaped HAp particles of 20–100 μm were produced. This process resulted in the presence of minor element such as Mg and Na and trace elements including Zn, Sr, and Ba in the sample [22]. Prior research has shown that minor and trace elements can enhance the formation of new bone during in vitro and in vivo test [24, 25].

Ruksudjarit *et al.* used a combination of calcination with vibro-milling technique to extract the HAp from bovine bone [19]. The bone then was calcined at 800 °C for 3 h before multiple milling steps were used. Two steps of milling were used, namely ball milling (24 h) and vibro-milling for different times (0, 1, 2, 4, and 8 h). Ruksudjarit *et al.* reported that needle-like HAp with diameter <100 nm and Ca/P ratio of 1.66 was produced by using this combination of methods [19]. The results showed that a higher distribution density of needle-like HAp was obtained at longer vibro-milling times. Ruksudjarit *et al.* stated that the optimal vibro-milling time for producing needle-like HAp between 2-4 h; at 8 h, the HAp needles were fractured and were inhomogeneous in terms of size and shape. Therefore, they suggested that the vibro milling time longer than 8 h will break the HAp needle to finer particle with same diameter [19].

Londoño-Restrepo *et al.* used a combination of hydrothermal and calcination to extract HAp from bovine bone [23]. The hydrothermal method was employed to remove the excess fat and protein from the bone; afterwards, the powder was calcined at temperatures of 700 °C–1100 °C. The morphology of Hap changed with variations in the calcination temperatures (e.g., irregular at 700 °C and semi spherical at 800 °C). At temperatures exceeding 800 °C resulted in the formation of HAp dehydroxylate.

Aside from bovine bone, other mammalian bones such as camel, horse, and pig bone have been used to extract HAp. Calcination of camel bone resulted in nano-sized range from 79-97 nm pure crystalline HAp of irregular shapes [26, 27]. Jaber *et al.* subjected the bone to pretreatment followed by calcination at 1000 °C for 3 h [26] and milling using planetary ball mill at 250 rpm for 1 h. The resultant XRD results showed that the pure crystalline HAp was produced with Ca/P ratio of 1.66 which is close to the stoichiometric ratio. In addition EDS analysis revealed the presence of minor elements such as Mg and Na in the sample [26]. Cho *et al.* stated that the substitution of sodium for calcium in HAp enhanced the apatite-forming capacity in SBF as and exhibited excellent osteoconductivity compared to pure HAp [28]. Research by Rahavi *et al.* on the calcination of camel and horse bone at 700 °C for 2 h [27] resulted in the production of irregularly shaped nano-sized HAp. The size of the extracted HAp from the camel and horse bone were 97 nm and 28 nm respectively. The results showed that the calcination produced crystalline HAp with Ca/P ratio of 2.036 for camel and 2.131 for horse, both of which are higher than that seen for stoichiometric HAp [27]. Elemental analysis showed that besides calcium and phosphorus, traces elements such as Na, Mg, Sr, Fe, Al, and Zn were present in the calcined camel and horse bone. The presence of trace elements in the HAp can enhance and accelerate the growth of the bone [29].

Ofudje *et al.* used calcination to extract HAp from pig bone [30] where clean and dried pig bone were calcined at 600 °C, 800 °C, and 1000 °C prior to pre-treatment. The results showed that pure crystalline HAp was produced with a rod-like morphology (38–52 nm length and 7–13 nm width). The Ca/P ratio of the sample after calcining at 1000 °C was 1.88. Most biological apatite is non-stoichiometric owing to the presence of the trace elements that replace the Ca in the apatite lattice [31].

Janus *et al.* and Haberko *et al.* employed a combination of alkaline heat treatment and calcination to extract HAp from pig bone [31, 32]. The clean pig bone was treated in 4 M NaOH at 100 °C for 24 h; this process was repreated again using a new NaOH solution. Then, the samples were washed until the filtrate reached a pH 7 and then dried. Janus *et al.* used 800 °C and 1200 °C to calcine the samples [32]. The results showed that pure HAp phase was produced at 800 °C while at 1200 °C, a mixture of HAp and CaO were present. Research by Haberko *et al.* reported that at calcination temperatures higher than 700 °C, trace amounts of CaO were present. Both sets of researchers were able to extract HAp from the pig bone in nanometer size range from 70- 180 nm but the shapes of the particles were different. Janus *et al.* formed irregularly shaped HAp while Haberko *et al.* produced plate-like HAp. The Ca/P ratio of the sample from both works showed 1.709 [32] and 1.72 [33] Ca/P ratios which higher than the stoichiometric ratio owing to the presence of trace CaO in the extracted HAp. The summary of methods used for extraction of HAp from mammalian sources is presented in Table 1.

**Table 1:** Summary of methods used for extraction of HAp from mammalian sources.

Source	Method of extraction	Ca/P ratio	Crystalline phases	Particle size	Shape	Reference (s)
Bovine bone	Alkaline hydrothermal hydrolysis	1.86	HAp	Nanosize	Nanoflakes	[20]
	Subcritical water process	1.56	HAp	Nanosize	Nanoflakes	[20]
	Calcination	- 1.67 1.5 >1.67	HAp (900 °C) HAp (750 °C) HAp, β-TCP (850 °C) HAp, β-TCP	30 nm <420 μm 420–500 μm 20–100 μm	- - - Irregular	[18] [21] [22]
	Alkaline heat treatment	>1.67	HAp	20–100 μm	Irregular	[22]
	Calcination + vibro milling	1.66	HAp (800 °C)	<100 nm (<2 h)	Needle like	[19]
	Hydrothermal + calcination	-	HAp, HAp dehydroxylate	-	Irregular (700 °C) semi-spherical (800 °C)	[23]
Camel bone	Calcination	1.66	HAp (1000 °C)	79 nm- 0.9 μm	Irregular	[26]
		2.036	HAp (700 °C)	97 nm	Irregular	[27]
Horse bone	Calcination	2.131	HAp (700 °C)	28 nm	Irregular	[27]
Pig bone	Calcination	1.88	HAp (1000 °C)	38–52 nm length 7–13 nm width	Rod-like	[30]
	Alkaline heat treatment + calcination	1.709 (800 °C) 1.657 (1200 °C) 1.72	HAp (800 °C) HAp, CaO (1200 °C) HAp (700 °C) HAp, CaO (<700 °C)	70–180 nm (800 °C) and 200–700 nm (1200 °C) Nanometer range	Irregular Plate-like	[32] [33]

Table 1 shows that the calcination method was the most popular method for fabrication compared to others. The calcination process removes the organic constituent in the bone by the thermal process. The organic matter is converted to carbon dioxide and ash (calcium phosphate compounds) as per Eq. (1). This calcium phosphate usually does not decompose at temperature below 1200 °C and will be present as different calcium phosphate (CaP) phases. Increasing temperatures increases the crystallinity of the HAp particles along with other calcium phosphate phases such as β-TCP. On the other side, use of high calcination temperatures will remove all pathogens and prevent the possibility of transmission of diseases such as bovine spongiform encephalopathy and Creutzfeldt Jakob disease [19, 34, 35, 36]. Researches have concluded that pathogens cannot survive at temperature above 800 °C [19, 36].

(1) $$\text{Mammalian}\;\text{bone}\;\text{(Organic}\;\text{matter}\;+\;\text{HAp}\;+\;\text{water)}\overset{\;\text{Calcination}\;\;}{\to }\text{CaP}\;\text{phases}\;+\;{\text{CO}}_{2}$$

Other methods such as alkaline heat treatment have been used to extract HAp from mammalian bone. In this method, the alkaline solution usually NaOH is used to remove the organic matter from the bone. The NaOH solution hydrolyzes the organic component in the bone and the remaining calcium phosphate is rinsed and separated using filtration. However, alkaline heat treatment produces low crystallinity HAp compared to calcination. Sun *et al.* revealed that the crystallinity of HAp produced using alkaline heat treatment was much lower than that seen for calcined HAp [22].

In addition, mammalian bones contain higher source of ions and trace elements as reported in Table 2 [22,26,27,30]. As listed in Table 2, Mg and Na ions are the most frequently found ones in mammalian sources. Other trace elements such as Sr, Zn, and Al also have been observed. The concentration of these elements vary and this is owing to differences in the diet of the animals.

**Table 2:** Summary of the properties of HAp extracted using different method from mammalian sources.

	Methods		
Properties	Calcination method	Alkaline hydrolysis method	Combination method
Morphology	Irregular, rod-like	Flakes-like, irregular	Needle-like, irregular, plate-like
Particle size	28–900 nm	20–100 μm	70–700 nm
Crystallinity	High	Low	High
Ca/P ratio	1.500–2.131	1.67–1.86	1.657–1.720
Presence of trace element	• Mg, Na, Zn, Sr, Ba [22] • Mg, Na [26] • Mg, Na, Sr, Fe, Al, Zn [27]	• Mg, Na, Zn, Sr, Ba [22]	-

The Ca/P ratio of the extracted HAp from the mammalian bone has varied from 1.5 to 2.13. According to Table 2, the calcination method results in a high value of Ca/P value compared to alkaline hydrolysis while combination of methods produce a narrower range of Ca/P that is close to stoichiometry. According to Akram *et al.* the non-stoichiometric Ca/P ratio of the HAp arises the presence of trace elements [9] which replace Ca in the apatite lattice [31]. The use of high calcination temperatures can result in β-TCP phase formation from Hap which can reduce the Ca/P ratio. The presence of CaO can increase the Ca/P ratio. The aim of achieving Ca/P values close to stoichiometry since either the trace elements or β-TCP can enhance the properties of these materials for bone implant purposes.

**Fig. 2:**
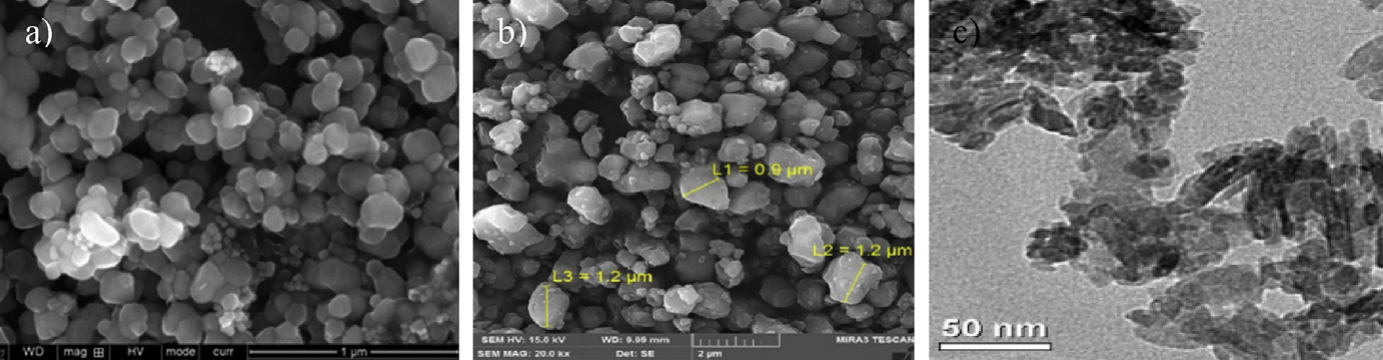
Micrographs of HAp extracted from mammalian sources using calcination. a) SEM micrograph of bovine bone calcined at 800 °C. (Adapted from [37], with permission from Elsevier). b) SEM micrograph of camel bone calcined at 1000 °C. (Adapted by permission from [26], Copyright, 2017). c). TEM micrograph of HAp synthesized from pig bone at 1000 °C. (Adapted from [30], with permission from Elsevier).

**Fig. 3:**
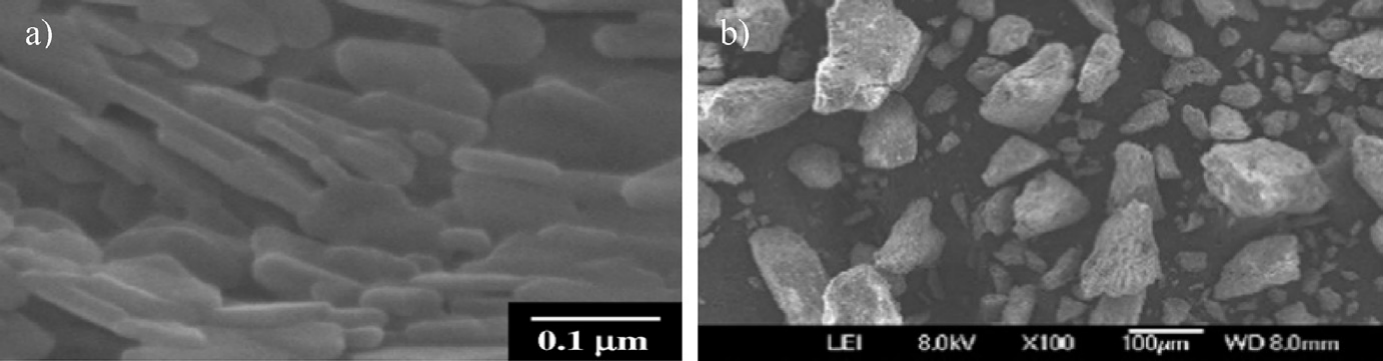
Micrographs of HAp extracted from mammalian sources using alkaline heat treatment method. a) FESEM micrograph of bovine bone. (Adapted from [38], with permission from Elsevier). b) SEM micrograph of bovine bone. (Adapted from [22], with permission from Elsevier).

The morphological analyses of the HAp extracted from the mammalian bone show that the particles are mostly irregular in shape, with some studies showing the presence of rods, flakes, needles, and plate-like shapes. Figs. 2 and 3 show SEM micrographs of HAp produced via calcination and alkaline hydrolysis, respectively. Fig. 3 shows that use of the same method and same source (bovine) can result in different shapes of HAp. Thus the shape variation is believed to not be affected by the method or source. For example, calcination of same source of bone could produce the various shapes of HAp such as rod-like, spherical, and needle like. In addition, the rod shape HAp could be produced using different extraction methods such as alkaline hydrolysis and combination method. It can be concluded that, there is no relation between the morphology of HAp with the extraction method and source.

The size of HAp obtained did not show any correlation with the extraction method. The use of additional milling helped to reduce the size of the HAp particles to the nanometric size which is close to that of human HAp. In addition, the nanosized particles have advantages in terms of high surface activity and ultrafine structures [39], higher bioactivity, and better resorbability than micron-sized particles [40, 41]; this also allows for improved sinterability and densification avoiding microcrack formation [42, 43] and lowering apoptotic cell death [40]. Tables 1 and 2 show the different size ranges of HAp produced from different sources and different methods.

#### 2.2.2 Aquatic or marine sources

The increasing consumption of fish around the world has caused significant increase in fish waste production in the form of scales and bones. The recovery of fish scales and bones allows for extraction of HAp and reduction in solid wastes in the fisheries industry [44]. The fish bone is rich in calcium, phosphate, and carbonate which make it a great source for extraction of HAp [9].

The pretreatment of the fish bones/scales has been done using different methods. Generally, pretreatment is done first to remove fleshy meat and unwanted debris on the bone and scale and one of the common method used is boiling [45, 46, 47]. The fish bone/scale is also pretreated by rinsing in tap water followed by soaking in low concentrations of hydrochloric acid (0.1–1 M HCl) for the deproteinise process and then washed in tap water [44, 48, 49]. Usually fish bones and scales were cut or ground into small pieces before further treatment.

Table 2 summarises the properties of HAp extracted from fish bone and scale. In short, the calcination of fish bone at increasing temperatures will increase the particle size of HAp [46, 47]. Sunil & Jagannatham reported that highly crystalline pure HAp was produced after calcination at 600°-1000 °C. The Ca/P ratio in the sample calcined at 600 °C was 1.63 which is slightly lower than the stoichiometric HAp. In addition, the EDS analysis of the sample calcined at 600 °C revealed the presence of Mg ions in the sample which act as micronutrients for metabolic activities in tissues [47]. Pal *et al.* calcined *Later calcarifer* bones at a wide range of calcination temperatures from 200°, 400°, 800°, 1000°, and 1200 °C for 1 h. However, Pal *et al.* produced HAp along with TCP at higher calcination temperatures (1200 °C) [46]. They found that the Ca/P ratio of the sample calcined at 1200 °C was 1.62 which was also slightly lower than stoichiometric HAp owing to TCP formation.

Furthermore, Venkatesan *et al.* performed a comparative study of calcination and alkaline hydrolysis for extraction of HAp from *Thunnus obesus* fish bone [50]. Venkatesan *et al.* calcined tuna fish bone at 900 °C for 5 h. In the alkaline heat treatment method, tuna fish bone was soaked with 2 M NaOH and heated at 250 °C for 5 h and then the precipitate was washed and adjusted to neutral pH using water and then dried. Both methods were able to produce pure HAp. However, the HAp produced from calcination showed higher crystallinity compared to the alkaline heat treatment. The calcination produced Hap with larger particle sizes compared to the alkaline heat treatment method. Ozawa & Suzuki stated that using the alkaline heat treatment can preserve the size of the crystals while these tended to agglomerate in thermal calcination [51]. In addition, Venkatesan *et al.* stated that the complete removal of organic moieties by thermal calcination results in the particles growing while alkaline heat treatment does not alter the size. However, the Ca/P ratio of HAp produced from calcination was 1.65 which is near to that of stoichiometric HAp while the ratio produced by alkaline heat treatment was higher.

Besides fish bone, the scales also can be used as a HAp source. Mondal *et al.* and Paul *et al.* carried out calcination using fish scales. Paul *et al.* conducted calcination at 200°, 400°, 800°, 1000°, and 1200 °C for 1 h. When calcination was done at 800 °C, pure HAp was obtained while calcining at temperatures >1000 °C resulted in the formation of trace amounts of β-TCP with the amounts increasing at 1200 °C. The ICP-AES analyses showed that the HAp derived from fish scale contain several trace elements including Mg, Sr, Na, and K. Previous researchers showed that the apatite structure subtituted by Sr can enhance its bioactivity [53]. In other research, Mondal *et al.* calcined fish scales at 1000 °C and then milling was done using a pulvarized mill for 16 h to produce pure HAp phase with Ca/P ratio of 1.71. The morphological structure revealed nearly spherical shapes with 76.62 nm average size [52].

Panda *et al.* extracted HAp using a combination of alkaline heat treatment and calcination [44] resulting in pure crystalline HAp with agglomerates of average size 76.62 nm and Ca/P ratio of 1.62. In this method, the fish scale was first treated with 1 N NaOH and then treated with calcium chloride dihydrate (CaCl_2_.2H_2_O) at 75 °C to treat the deficiency of calcium in the fish scale, followed by calcination at 800 °C for 1 h.

Pon-On *et al.* employed alkaline hydrolysis to extract HAp from fish scales [48]. Compared to other alkaline hydrolysis treatments, Pon-On *et al.* pretreated the scale by soaking in NaOH solution, followed by filter pressing to form a cake which was sealed in a plastic bag before boiling in distilled water at 100 °C for 30 min. This was then stored at -20 °C prior to use. The results showed the formation of flat-plate pure HAp with sizes ranges of 15–20 nm width and 100 nm lengths with Ca/P of 2.01. The higher Ca/P ratio was owing to the presence of carbonate ions that substituted for phosphates in the apatite structure (B-type carbonate HAp) and this was confirmed by the FTIR results. Kongsri *et al.* also used alkaline heat treatment for the extraction of HAp from fish scales [49] with two stages of treatment with a first step of 5% NaOH treatment for 5 h. The treated fish scale was then filtered and dried before treatment with 50% NaOH for 1 h. The treated powder was then filtered and washed with deionized water until the pH was neutral and then dried. The method produced nano-sized high purity HAp with Ca/P ratio of 1.66. The HAp produced by alkaline heat treatment [48, 49] are less crystalline than the HAp produced by calcination. According to Alparslan *et al*, low-crystalline HAp has higher biodegradation rate and is more metabolically active compared to high-crystalline HAp [54]. The alkaline heat treatment produced smaller particle sizes of HAp compared to calcination.

It was also reported that ionic liquid pretreatment method produces pure HAp with slightly large particle size of 1870 nm with variation in surface morphology [55]. In this method, the ground fish scale powder reacted with 1- butyl-3-methylimidazolium acetate and was later boiled at 100 °C for 12 h. The equal volume of reaction mixture was then mixed with 0.5 M NaOH until the pH was 9 and the precipitate of HAp formed was separated by centrifugation at 11000 rpm for 30 min. According to Meng *et al.* the collagen part will interact with ionic liquid through hydrogen bonding and cause dissolution [56]. In addition, the inorganic part of HAp will remain undissolved and was obtained as a precipitate [55].

On the other hand, Huang *et al.* used enzymatic hydrolysis to extract HAp from fish scales [57]. Two types of enzymes, protease and flavourzyme were used to hydrolyse the protein component in the fish scale such as collagen which is the major protein. The fish scale was first hydrolysed using 1% protease for 2.5 h and 0.5% flavourzyme for another 0.5 h. They successfully produced HAp with slightly high Ca/P ratio of 1.76. The pure HAp phase produced was irregular in shape with average sizes of 719.8 nm [57]. The samples were seen to have broad XRD peaks reflecting the low crystallinity of the HAp phase.

Table 3 shows that many methods have used to extract HAp from fish scales while calcination and alkaline heat treatment were used to extract from fish bones. The physical physiology of the fish scale in terms of greater flexibility (contains more collagen compared to bone) and softer compared to the bone allows for easier extraction from other methods. Ionic liquid pretreatment and enzymatic hydrolysis have been used in addition to calcination and alkaline hydrolysis.

**Table 3:** Summary of methods used for extraction of HAp from aquatic or marine sources.

Source	Method of extraction	Ca/P ratio	Crystalline Phases	Particle size	Shape	Reference (s)
Fish scale	Calcination	1.71 (1200 °C)	HAp (800 °C) HAp, β-TCP (≥1000 °C)	30 nm	Irregular	[45]
		1.71	HAp (1000 °C)	76.62 nm	Nearly spherical	[52]
	Alkaline heat treatment + calcination	1.62	HAp	76.62 nm	Agglomerate	[44]
	Alkaline heat treatment	2.01	HAp	15–20 nm width 100 nm length	Flat-plate	[48]
		1.66	HAp	15–20 nm	Hexagonal	[49]
	Ionic liquid pretreatment	1.60	HAp	1870 nm	Vary	[55]
	Enzymatic hydrolysis	1.76	HAp	719.8 nm	Irregular	[57]
Fish bone	Calcination	1.62 (1200 °C)	HAp, TCP (present at high temperature)	5–55 nm	Irregular	[46]
		1.63	HAp (1000 °C)	64.5–330 nm	Agglomerate	[47]
		1.65	HAp (900 °C)	0.3–1.0 μm	Irregular	[50]
	Alkaline heat treatment	1.76	HAp	5–10 nm width 17–71 nm length	Rod like	[50]

As calcination and alkaline hydrolysis methods have been detailed in Section 2.2.1, ionic liquid pretreatment and enzymatic hydrolysis will be further elaborated in this section. In ionic liquid pretreatment, the ionic liquids contain high hydrogen bond basicity value which can dissolve the biopolymers such as cellulose or collagen [55, 58]. Thus, during treatment, the interaction of collagen in the fish scale with the ionic liquid through hydrogen bonding will cause the dissolution of the organic part (collagen) while the inorganic part (HAp) will remained undissolved and be obtained as a precipitate [55]. Thus HAp was separated through filtration and evaporation.

In enzymatic hydrolysis method, the enzyme was used to hydrolyse the organic component in the scale. In order to hydrolyse the collagen, a specific enzyme must be used during the treatment. As collagen is a protein component, protease enzyme is used to hydrolyse the collagen. The enzymes usually work best at their optimal temperature and pH. As all the collagen was degraded, the mixture containing the enzyme will be subjected to high temperatures in order to deactivate the enzyme and the remaining HAp will be collected using centrifugation [57].

Use of the hydrolysis methods (alkaline hydrolysis, enzymatic hydrolysis) and ionic pretreatment method can allow for extraction of pure HAp without transforming the HAp to other CaP phases. For instance, the use of calcination may alter the HAp phases into β-TCP. The hydrolysis only removes the organic component of the bone or scale without altering the chemical structure of HAp. In term of crystallinity, the use of calcination produces phases of higher crystallinity compared to other methods.

**Fig. 4:**
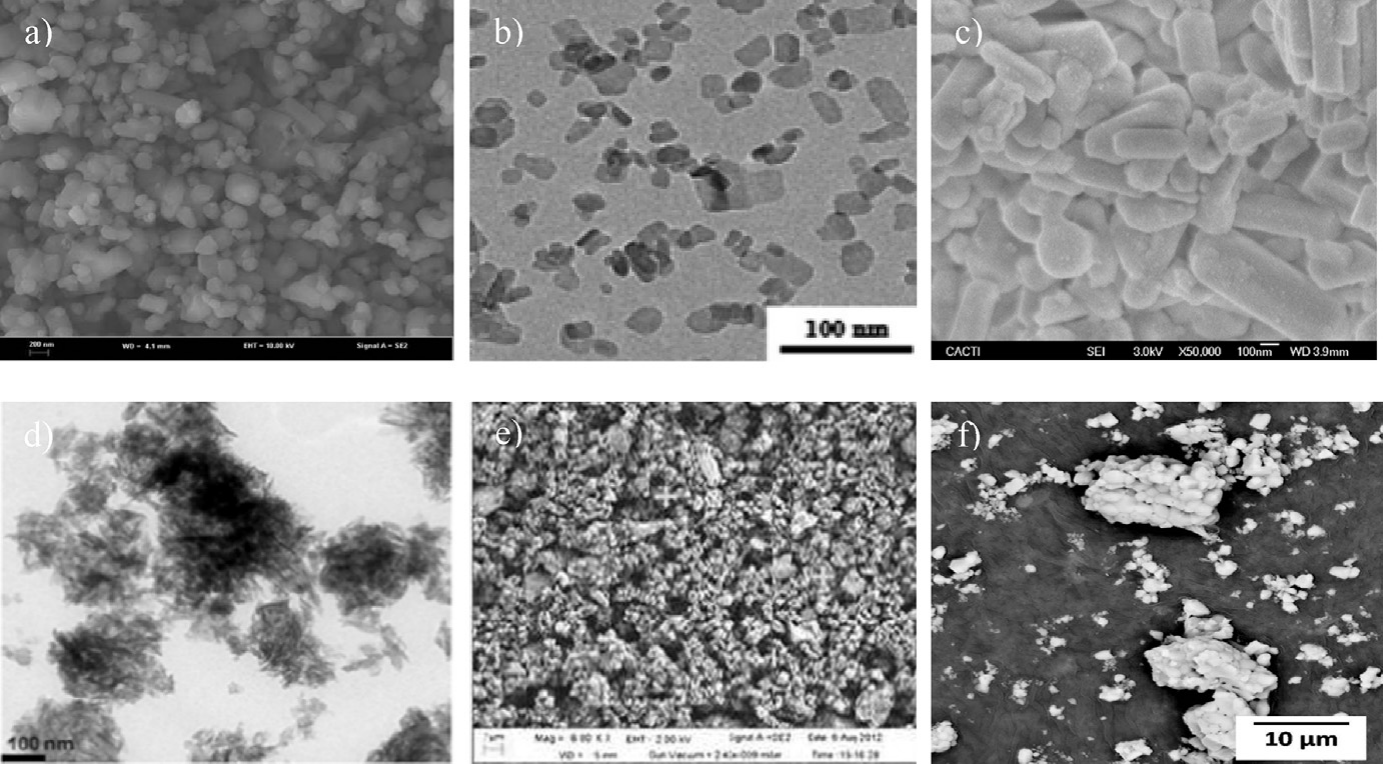
Micrographs of HAp extracted from aquatic or marine sources. a) SEM micrograph of fish bone calcined at 900 °C. (Adapted from [59], with permission from Elsevier). b) TEM micrograph of fish scale extracted using alkaline heat treatment. (Adapted from [49], with permission from Elsevier). c) SEM micrograph of fish bones calcined at 600 °C. (Adapted from [35], with permission from Elsevier). d) TEM micrograph of fish scale extracted using alkaline heat treatment. (Adapted from [48], with permission from Elsevier). e) FESEM micrograph of fish scale calcined at 1000 °C. (Adapted from [52], with permission from Elsevier). f) SEM micrograph of fish scale calcined at 1200 °C. (Adapted from [45], with permission from Elsevier).

When analyzing the morphology of the extracted HAp from aquatic and marine sources, the shapes of HAp were seen to be irregular, nearly spherical, flat plates, rod-like and so on. Fig. 4 shows SEM micrographs of the HAp extracted from these sources using calcination. Different morphologies were formed using the same method (calcination). From Tables 3 and 4 it can be concluded that the shape of the HAp is not influenced by the method or sources. Table 4 shows that by using calcination and hydrolysis, particles in the size range from 5 nm to 1000 nm could be successfully produced. On the other hand, the ionic liquid pretreatment method results in large particles of size 1870 nm.

**Table 4:** Summary of the properties of HAp extracted using different method from aquatic or marine sources.

	Methods			
Properties	Calcination method	Hydrolysis method (alkaline and enzymatic)	Ionic liquid pretreatment	Combination method
Morphology	Irregular, nearly spherical, agglomerate	Flat-plate, hexagonal, rod-like, irregular	Vary	Agglomerate
Particle size	5–1000 nm	5.0–719.8 nm	1870 nm	76.62 nm
Crystallinity	High	Low	Low	High
Ca/P ratio	1.62–1.71	1.66–2.01	1.60	1.62
Presence of trace element	• Mg [47] • Mg, Sr, Na, K [45]	-	-	-

The Ca/P ratio of the extracted HAp was not similar to that of stoichiometric HAp. As summarized in Table 4, using calcination, the Ca/P ratios were near to the stoichiometric HAp being in the range 1.62–1.71. Otherwise, using hydrolysis, the Ca/P ratio can be increased to 2.01. According to this study, the higher Ca/P ratio obtained in fish scale HAp was due to the presence of carbonate ions substituting for phosphate in the apatite structure (B-type carbonate) as confirmed by the FTIR spectrum [48].

In addition, the elemental analysis has confirmed the presence of trace elements in the HAp extracted from fish scales and fish bones produced using calcination. Similar to that case of mammalian sources, Mg ions were present in Hap from both fish bone and scale, while trace element such as Sr, Na, and K were found in fish scale. The HAp extracted from natural sources contains trace elements that help to enhance the properties of the HAp.

In conclusion, the calcination methods may transform HAp to other calcium phosphate phases such as β-TCP at high temperatures. However, the alkaline heat treatment method, ionic liquid pretreatment, and enzymatic hydrolysis method produced pure phase HAp but with lower crystallinity compared to calcination method. In general, most of the methods listed in Table 2 could produce nano-sized HAp with different morphologies.

#### 2.2.3 Shell sources

The shells of cockle, clam, and egg are rich with calcium carbonate (CaCO_3_) which can be converted to HAp. Eggshell contains 94% CaCO_3_ which can be a source of Ca for HAp production [9]. These shells are abundant in nature and are generally discarded.

Generally, the shells were washed with distilled water to remove sand and dirt and further cleaned using bleaching agent. The shells then are calcined to form CaO [60] which along with excess of CaCO_3_ will be used for further treatment with chemicals to produce high purity HAp. Table 3 summarises the properties of HAp extracted from shells.

Mustaffa *et al.* carried out calcination and sol-gel precipitation method for the extraction of HAp from cockle shells [60]. The cleaned cockle shell was crushed and milled to form powder which was calcined at 600 °C to convert the CaCO_3_ to CaO. Then, the CaO powder undergoes sol-gel precipitation method to synthesise HAp. In sol-gel technique, the CaO powder is mixed with orthophosphoric acid and then ammonia solution, NH_4_OH is added to this to maintain it in alkaline condition for 24 h. The precipitate formed is rinsed and filtered before drying and further milling was done followed by sintering at 1150 °C. The results showed the formation of HAp which was spherical in shape of sizes 4.03–10.4 μm and Ca/P ratio of less than 1.68.

Santhosh & Prabu produced HAp from sea shells using calcination and wet chemical precipitation method [61]. In this method, the sea shell was first calcined at 900 °C to convert into to CaO phase after which 0.6 M H_3_PO_4_ was added drop-wise at the rate of 1 mL/min with continuous stirring. The pH was adjusted to 10 using ammonia solution. The precipitate was then washed with distilled water and dried before calcining at different temperatures (250 °C, 500 °C, 750 °C, and 1000 °C) for 2 h. This shows that process resulted in HAp with 1.8 [61]. The pure crystalline HAp was formed at 250 °C while at higher temperatures, β-TCP was formed. This method also produced nano-sized particle with 101 nm size with rod-like shapes. The chemical reactions involved during the calcination and precipitation methods are presented in Eqs. (2), (3), and (4).

(2) $${\text{CaCO}}_{3}\overset{900^{\circ }\text{c}\;}{\underset{\Delta }{\to }}\text{CaO}\;+\;{\text{CO}}_{2}$$

(3) $$\text{CaO}\;+{\;\text{H}}_{2}\text{O}\to \text{Ca(}{\text{OH}}_{2}\text{)}$$

(4) $${10\;\text{Ca(}{\text{OH}}_{2}\text{)}\;+\;6{\;\text{H}}_{3}{\text{PO}}_{4}\to {\text{Ca}}_{10}\text{(}{\text{PO}}_{4}\text{)}6\text{(OH)}}_{2}\;+\;18{\;\text{H}}_{2}\text{O}$$

Mohamad Razali *et al.* applied different calcination temperatures (450 °C and 800 °C) to calcine the cockle shell followed by the hydrothermal process to produce HAp [62]. The calcined cockle shell was composed of CaCO_3_ which was further treated with di-ammonium hydrogen phosphate, ((NH_4_)_2_HPO_4_) and water to convert the CaCO_3_ to HAp at 5:3:1 molar ratio of CaCO_3_: (NH_4_)_2_HPO_4_: H_2_O. The ammonia solution was added to adjust the pH of the mixture to 10.5 and the mixture was subjected to hydrothermal reaction at 100 °C for 1 h under continuous stirring. The mixture was then rinsed and filtered before drying to obtain HAp powder. The chemical reaction of HAp formation can be expressed in Eq. (5).

(5) $${{{10\;{\text{CaCO}}_{3\;}+6\;\text{(}{{\text{NH}}_{4}\text{)}}_{2}{\text{HPO}}_{4}\;+2{\;\text{H}}_{2}\text{O}\to {\text{Ca}}_{10}\text{(}{\text{PO}}_{4}\text{)}}_{6}\text{(OH)}}_{2}\;+6\;\text{(}{\text{NH}}_{4}\text{)}}_{2}{\text{CO}}_{3}\;+4{\;\text{H}}_{2}{\text{CO}}_{3}$$

XRD analyses showed that the sample calcined at 450 °C produced a combination of HAp and calcite, while pure HAp was produced during calcining at 800 °C. The existence of calcite peak was owing to incomplete conversion of calcite to HAp at 450 °C. This method resulted in rod-like shapes with 207 nm length and 27 nm diameter [62].

Pal *et al.* reported that a combination of calcination and mechanochemical treatment was used to extract HAp from clam shells [63]. In this method, the crushed clam shells were calcined at 1000 °C for 3 h to convert the CaCO_3_ to CaO. The CaO and H_3_PO_4_ was milled for 20 h at 300 rpm. In this research, different weight ratios of CaO: H_3_PO_4_ were studied (1 : 0.75, 1 : 1.25, 1 : 1.5, and 1 : 1.75). Lastly the powder was further calcined at 1000 °C for another 3 h. The XRD results showed that as the weight ratio of CaO:H_3_PO_4_ increased, the HAp phase also increased. The pure crystal HAp was produced at a weight ratio of CaO:H_3_PO_4_ of 1:1.5 with crystal size of 53 nm. Use of other proportions of CaO:H_3_PO_4_ produced other CaP phases with sizes ranging from 58-67 nm [63]. The chemical reactions of calcination and mechanochemical method to produce HAp from the clam shell was presented in Eqs. (6), (7), and (8) [63].

(6) $${\text{CaCO}}_{3}\overset{1000\text{°C}}{\underset{\Delta }{\to }}\text{CaO}\;+\;{\text{CO}}_{2}$$

(7) $$2\;\text{CaO}\;+{\;\text{H}}_{3}{\text{PO}}_{4}\to {\text{CaHPO}}_{4}\;+\;\text{Ca(}{\text{OH}}_{2}\text{)}$$

(8) $${{5\;{\text{CaHPO}}_{4}\;+\;5\;\text{Ca(}{\text{OH}}_{2}\text{)}\;+{\;\text{H}}_{3}{\text{PO}}_{4}\overset{1000\text{°C}\;\;}{\underset{\Delta }{\to }}{\text{Ca}}_{10}\text{(}{\text{PO}}_{4}\text{)}}_{6}\text{(OH)}}_{2}\;+\;8{\;\text{H}}_{2}\text{O}$$

Goloshchapov *et al.* used a combination of calcination and wet precipitation using eggshells [64]. First, the egg shell was calcined at 900 °C for 2 h. The obtained CaO was mixed with distilled water at 100 °C that transforms it to calcium hydroxide. The calcium hydroxide was then titrated with 0.6 M orthophosphoric acid [64] and then the precipitate was calcined at 400 °C, 700 °C, and 900 °C for 1 h. The results stated that the HAp produced at different pH showed different Ca/P ratios. Therefore, as the pH increased, the ratio of Ca/P increased. The lower pH will decrease the Ca/P ratio and is closer to the stoichiometric ratio.

Neelakandeswari *et al.* used precipitation methods for synthesis of HAp from eggshells [65]. First, 1 mmol of calcium chloride dihydrate (CaCl_2_.2H_2_O) was used to adjust to several pH (9, 10, 11). Then, diammonium hydrogen phosphate ((NH_4_)_2_HPO_4_) and egg shell membrane (ESM) was added to the calcium chloride dihydrate (CaCl_2_.2H_2_O) solutions of different pH. The mixture was stirred for 24 h before washing and centrifugation to obtain the precipitate which was dried to obtain HAp powders. The results showed that this method produced needle-like shapes with 5 nm diameter and 50–100 nm length at pH 9. However, the Ca/P ratio produced by this method is 1.425 which is much lower than that of stoichiometric HAp. Even though all samples showed the formation of HAp, the XRD peaks were broad which indicates the low crystallinity of the sample [65]. Maybe, there are presences of amorphous calcium phosphate, ACP in the HAp sample. According to literature ACP have Ca/P value range from 1.2-2.2.

Shavandi *et al.* synthesised HAp from mussel shells [66] using rapid microwave irradiation. This method was able to produce high amounts of HAp by using less energy and at a faster rate. First, the cleaned muscle shell was calcined at 900 °C for 30 min to produce CaO which was ground and mixed with 0.1 M EDTA to form a solution of Ca-EDTA complex. Then, 0.06 M Na_2_HPO_4_ was added dropwise at rate of 4 mL/min and then stirred for 15 min. The pH was adjusted to 13 before treatment was done in the microwave. The obtained powder was further dried using a vacuum oven followed by further calcination at 650 °C for 1 h. The results showed that this method produced pure crystalline HAp with Ca/P ratio of 1.65 which is near to that of stoichiometric HAp and consisted of rod-like nano sized HAp (30–70 nm).

In summary, the shells are a rich source of CaCO_3_ and can enable the extraction of HAp. However, it requires more synthesis steps to produce high purity HAp. Most of the methods described in Table 3 used a combination of methods to synthesis HAp. The first step involves conversion of CaCO_3_ in the shell to CaO first followed by reaction with a phosphate source such as phosphoric acid, ammonia solution or diammonium hydrogen phosphate and then adequate treatment to convert those calcium and phosphate source into hydroxyapatite. The treatment methods includes chemical (chemical precipitation), mechanical (mechanochemical) and thermal reaction.

The crucial part in order to extract HAp from this shell source is the usage of the appropriate molar ratio of phosphate source to that of the calcined shell (CaO) to produce the optimal amount of HAp. Otherwise, the extracted HAp will not be pure and can contain traces of CaO during the treatment. Mohamad Razali *et al.* shows the incomplete conversion of the CaCO_3_ to CaO at 450 °C results in the presence of trace calcite compound in the extracted HAp at the end of extraction.

**Fig. 5:**
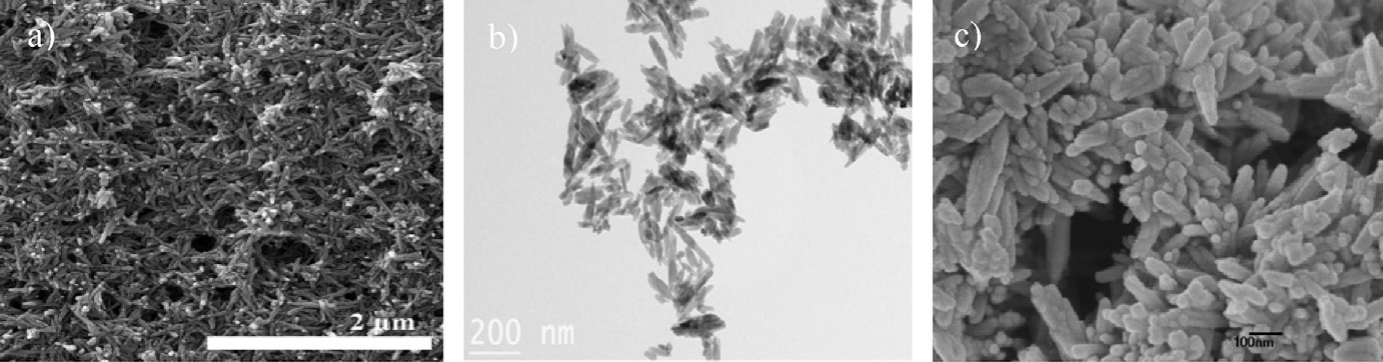
SEM micrographs of the HAp extracted from different sources using a combination of methods. a) SEM micrograph of HAp extracted from egg shells extracted using combination of calcination and wet chemical precipitation method. (Adapted from [67], with permission from Elsevier). b) TEM micrograph of HAp extracted from sea shells extracted using a combination of calcination and wet chemical precipitation method. (Adapted from [61], with permission from Elsevier). c) SEM micrograph of HAp extracted from mussel shells extracted using a combination of calcination and microwave irradiation treatment method. (Adapted from [66], with permission from Elsevier).

The results in Table 5 show that the extraction of HAp from shell sources produces particles of different morphologies. However, most of the methods were able to synthesise rods and needle-like HAp in addition to spherical and globular shape. Fig. 5 shows several micrographs of the HAp extracted from shell sources. As shown, all of the HAp particles have rod-like shapes. Although most of the extracted HAp showed the same pattern of shapes (needle/rod); however, this cannot be a foregone conclusion. In addition, most of the extracted HAp have sizes below 500 nm except for that obtained from HAp cockle shell using calcination and sol-gel precipitation method which had sizes of 4.03–10.40 μm.

**Table 5:** Summary of methods used for extraction of HAp from shell sources.

Source	Method of extraction	Ca/P ratio	Crystalline Phases	Particle size	Shape	Reference (s)
Cockle shell	Calcination + Sol-gel precipitation	<1.68	HAp	4.03–10.4 μm	Spherical	[60]
	Calcination + hydrothermal	1.76 (450 °C)	HAp, calcite	458 nm length 24 nm diameter	Needle-like	[62]
		1.8 (800 °C)	HAp	207 nm length 27 nm diameter	Rod-like	[62]
Clam shell	Calcination + mechanochemical	1.6	HAp	53–67 nm	Agglomerate polygonal	[63]
Sea shell	Calcination + chemical precipitation	1.8	HAp (250 °C) HAp + βTCP (500°, 750°, 1000 °C)	101 nm	Rod-like	[61]
Egg shell	Calcination + precipitation	1.7–2.1	HAp	30 nm	Globules	[64]
	Precipitation	1.425	HAp	5 nm diameter 50–100 nm length	Needle-like	[65]
Mussel shell	Calcination + rapid microwave irradiation	1.65	HAp	30–70 nm	Rod-like	[66]

In term of crystallinity, most of the HAp extracted using calcination has high crystallinity. However, Neelakandeswari *et al.* used precipitation to produce HAp from eggshells with low crystallinity compared to that obtained from calcination. The heat in calcination increases the crystallinity of HAp compared to precipitation. The extracted HAp produced from the shell source was non-stoichiometric. Most of the HAp extracted from combination have Ca/P ratio higher than 1.67 while the HAp extracted from precipitation had values lower than 1.67.

#### 2.2.4 Plant and algae sources

Research had revealed that plant and algae have the potential as sources of HAp. Various studies had been intensively conducted using plant parts (leaves, stalk, flower and wood), plant wastes (fruit peel), effluents from fruit processing industry, and also algae to extract HAp. Although the characteristics of the obtained HAp such as morphology, size and Ca/P ratio was not studied by some of the research, however, the potential of plants to produce HAp have been proven by XRD analysis.

Plant waste is one of the sources that have been used for the extraction of HAp. Orange and potato peel are fruit wastes that have been used for extraction of HAp [68]. Nayar & Guha used papaya leaves and Calendula flower for HAp extraction. Chemical precipitation was used to extract HAp from these fruit wastes and plant parts. The fruit waste or plant part was boiled in water for 10 min and the filtrate was mixed with 0.4 M alkaline calcium nitrate tetrahydrate solution and incubated for 24 h at 30 °C. After that the resulting solution was added to 0.156 M alkaline diammonium hydrogen phosphate solution, stirred and aged for one week at 30 °C. Finally the precipitate was washed with dionized water and dried and the XRD results showed the formation of HAp. The morphology of the HAp particles varied with the processing method and it is clear that biomolecules in the plant waste are important in controlling the synthesis of HAp [68]. Biomolecules such as enoic acids in potato peel extract, beta-carotene and vitamins present in the papaya leaf extract and limonene in orange peel play important roles in controlling the morphology and the size of the HAp particles. Thus, the HAp extracted from different plant wastes contain different biomolecules and consequently has different morphologies and sizes.

Shaltout *et al.* used the components of the leaves and stalks of selected plants (*Catha edulis*, basil, mint, green tea and trifolium) to extract HAp [69]. All the plants were calcined at temperatures of 600 °C, 700 °C, and 800 °C. The XRD results showed that out of the five plants, only *Catha edulis* and basil showed the presence of HAp phase after being calcined at 800 °C. The *Catha edulis* was able to produce high purity HAp phase while the basil plant produced a mixture of HAp and small amounts of Ca(OH)_2._ The XRD patterns also showed that increasing of calcination temperatures increased the crystallinity of the HAp particle. Furthermore, Govindaraj & Rajan also used *Moringaoleifera* leaves to extract HAp [70] using rapid microwave irradiation technique. First, the powdered leaves were treated with concentrated HNO_3_ and irradiated in a microwave at 50 W for 2 min to remove the organic components, and then the powder was washed and dried. The dried powder was then mixed with 0.1 M EDTA and 0.06 M (NH_4_)_2_HPO_4_ by sonication for 30 min. The pH was adjusted to 10 before irradiation in a microwave at 100 W for 5 min. Finally the white precipitate was washed and dried and high purity HAp was produced. In addition, the HAp extracted from *Moringaoleifera* leaves was in a nano size with flake-like morphology. The EDX analysis of the HAp particle showed that the particles had a Ca/P ratio of 1.72.

Besides leaves and stalks, the wood part of the plant can also be used to extract HAp but this process has not been investigated in detail owing to the complexity of the process. Tampieri *et al.* used a combination of five steps to achieve complete phase conversion of the rattan and pine wood into pure HAp [71]. The steps include pyrolysis, carburization, oxidation, carbonation, and phosphatization. Each step allows the phase transformation of the chemical components in the wood step by step until it produces pure phase HAp. In the first step, pyrolysis of the lignocellulose material is done to convert the organic substance into carbon. The second step which is carburisation involves transformation of carbon to calcium carbide (CaC_2_). The third step is oxidation where calcium carbide is converted to calcium oxide (CaO). Then the calcium oxide is transformed into calcium carbonate (CaCO_3_) by carbonation. In the last step, the calcium carbonate is converted into HAp in the phosphatization process. All in all, it requires many phase transformations to transform wood into the HAp phase [71]. Summary of the equations for the HAp transformations are stated in Eqs. (9), (10), (11), and (12):

(9) **Carburisation:** 2 C + Ca→CaC_2_

(10) **Oxidation:** 2 CaC_2_ +5 O_2_→2 CaO +4 CO_2_

(11) **Carbonation:** CaO + CO_2_→CaCO_3_

(12) **Phosphatization**_:_ 10 CaCO_3_ + 6 KH_2_PO_4_ + 2 H_2_O → Ca_10_(PO_4_)_6_(OH)_2_ + 6 KHCO_3_ + 4 H_2_CO_3_

Other than that, the abundance of phosphorus in wastewater of potato processing industry has allowed researchers the opportunity to recover phosphorus for the production of HAp [72]. Compared to other methods of synthesising HAp, research by Monballiu *et al.* used the combination of biological treatment and chemical treatment to produce HAp from potato waste effluent. In the biological treatment, the effluent of the potato processing industry was added to a nitrifying sludge into a bioreactor and treated in aerobic condition. Then, the effluent from the first reactor was treated with CaCl_2_ at a certain pH to initiate precipitation. Research showed 70% phosphate removal from the effluent at pH 7.83 where the yield of HAp was dependent on the availability of the Ca^2+^ ions. In summary, the usage of effluent that is rich in phosphorus from the potato processing industry has great potential for the production of HAp.

Besides plant and animal sources, HAp can be extracted from algae. Several species of algae have been used as a source to extract HAp such as microalgae *Scenedesmus* sp. and red algae *C. officinalis*. Teymouri *et al.* recovered the phosphorus present in *Scenedesmus* sp. to produce HAp and dittmarite via two different pathways [73]. The HAp was recovered using the hydrothermal mineralisation process. During the hydrothermal mineralisation pathway, calcium hydroxide was added to achieve a molar ratio of Ca/P equal to 1.67. In addition, synthetic pure HAp was seeded to the medium to facilitate the crystallisations of the HAp. The XRD results showed that without the seeding material, only whitlockite, Ca_18_Mg_2_(HPO_4_)_2_(PO_4_)_12_ is present. With the presence of seeding materials, HAp crystallisation is enhanced [74, 75, 76]. Thus, from the XRD results, HAp was the main phase present in the precipitates and a few peaks corresponding to whitlockite was also present. Magnesium contained in the microalgae and the HPO_4_^2−^ which is the predominant phosphate in algal hydrolysate favoured whitlockite formation [76]. Thus, the seeding of synthetic HAp is needed to facilitate HAp crystallization from the algal hydrolysate.

In another research, red algae *C. officinalis* was used for the extraction of HAp [77]. The red algae underwent pyrolysis at 650 °C and 700 °C to remove the organic materials followed by the alkaline hydrothermal in the pH range of 10–11. The XRD results showed that at 650 °C, the sample was converted to HAp, CaCO_3_, and β-TCP phases. The hydrothermal treatment was done by mixing the pyrolysed algae with ammonium di-hydrogenphosphate followed by stirring at 100 °C for 12 h to form HAp along with CaCO_3_ and β-TCP. At 700 °C, most materials were converted to HAp and a small amount of β-TCP. The summary of various methods used for extraction of HAp from plant and algae sources is presented in Table 6.

**Table 6:** Summary of the properties of HAp extracted from various plants and algae sources.

Source	Method of extraction	Ca/P ratio	Crystalline phases	Particle size	Shape	Reference (s)
*Moringaoleifera* leaves	Rapid microwave irradiation	1.72	HAp	Nano-size	Flakes	[70]
Wood: rattan and pine wood	Pyrolysis + hydrothermal	-	HAp	-	-	[71]
Potato peel	Chemical precipitation	-	HAp	250–500 nm	Cluster	[68]
Papaya leaves	Chemical precipitation	-	HAp	-	-	[68]
Orange peel	Chemical precipitation	-	HAp	50 nm	Rectangular	[68]
Calendula flower	Chemical precipitation	-	HAp	-	Elongate	[68]
Wastewater of potato processing	Chemical precipitation and biological pretreatment	1.53	HAp	-	-	[72]
*Catha edulis* (Khat)	Calcination	-	HAp (800 °C)	-	-	[69]
Basil	Calcination	-	HAp + Calcium hydroxide (800 °C)	-	-	[69]
*Scenedesmus* sp. microalgae	Hydrolysis-hydrothermal mineralization	-	HAp + Whitlockite	-	-	[73]
*C. officinalis* red algae	Pyrolysis + alkaline hydrothermal	-	HAp, CaCO_3_,βTCP (650 °C) HAp, βTCP, SiO_2_ (700 °C)	-	-	[77]

**Fig. 6:**
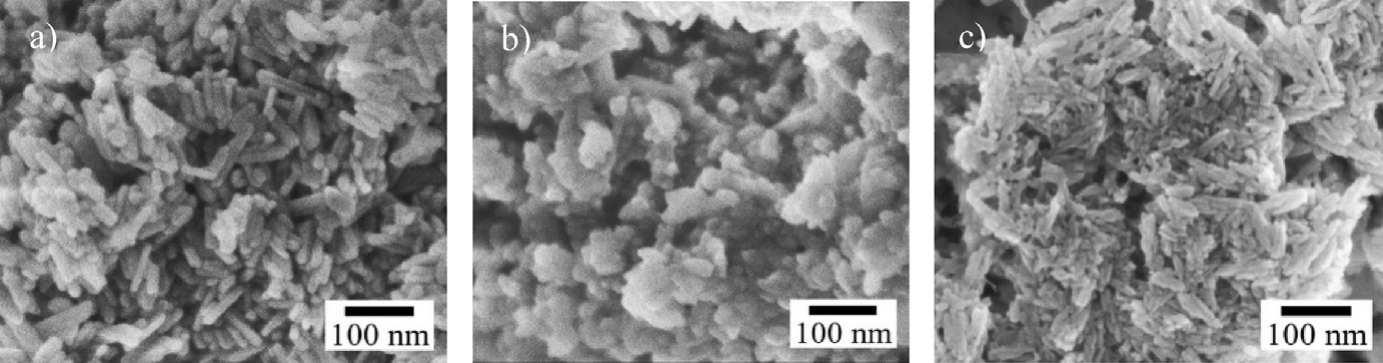
SEM micrographs of HAp extracted from plant sources using different methods. a) SEM micrograph of HAp extracted from a mixture of egg shell and pomelo peel using hydrothermal method. (Adapted from [78], with permission from Elsevier). b) SEM micrograph of HAp extracted from a mixture of egg shell and grape peel using hydrothermal method. (Adapted from [78], with permission from Elsevier). c) SEM micrograph of HAp extracted from a mixture of egg shell and sweet potato peel using hydrothermal method. (Adapted from [78], with permission from Elsevier).

As listed in Table 6, different morphologies of HAp were produced from different sources of plant such as flakes, clusters, rectangular shapes, and elongates. In addition, Fig. 6 shows the micrographs of HAp extracted from a mixture of eggshell and fruit peel. As discussed above, the technique and source does not influence the shape of the extracted HAp owing to the absence of similarity in patterns when the same method and source were used. Table 6 shows that some of the HAp extracted from the plant was nano-sized (50–500 nm).

The XRD patterns show that the HAp was successfully extracted from some of the plant and algae sources. However, the algae source did not allow successfully conversion of all the CaCO_3_ to HAp. The result from Table 6 shows that trace compounds are present such as whitlockite, CaCO_3_, and β-TCP. Similar to the discussion in Section 2.2.4, the molar ratio of the added phosphate precursor is important in order to enable complete conversion of the CaCO_3._ The β-TCP is formed from the degradation of the HAp at high calcination temperatures. According to Jang *et al.* the presence of magnesium will inhibit apatite formation and form whitlockite [79]. Due to the presence of magnesium in the central atom of chlorophyll in the algae, and the pH used during the reaction of the algal hydrolysate, all conditions favour the formation of whitlockite [73]. In term of crystallinity, the XRD pattern shows that the use of calcination or thermal processes will increase the crystallinity of the HAp while the chemical precipitation method results in low crystallinity HAp.

In summary, it is seen that plant and algae have some potential to be used as sources of HAp extraction. However, most of the methods required the addition of calcium or phosphate precursor because of the lack of one of these components in the plant itself. Thus, further steps are required to transform the phase into the pure HAp phase. This complicated process and the usage of additional calcium or phosphate source will make favour the use of plants for HAp extraction. For this reason, not much research has focussed on extraction of HAp from plant sources.

#### 2.2.5 Mineral sources

Among the mineral sources that contain calcium, limestone is one of the natural minerals used for synthesising hydroxyapatite. Naturally, limestone is formed from the precipitation of the animal shells or skeletons, foraminifera or algae which are rich in CaCO_3_ [80]. The abundance of limestone makes it one of the possible calcium sources to be used for synthesising of hydroxyapatite.

There are several methods that have been used to convert CaCO_3_ into HAp. Most of them used combination of method. As seen in Table 5, the limestone will be calcined first before another treatment such as precipitation, hydrothermal or ultrasonic irradiation is used. The purpose of calcination is to convert the CaCO_3_ phase to CaO [82, 83]. Jamarun *et al.* used calcination at 900 °C while Klinkaewnarong & Utara used 800° to 1100 °C, both studies used 5 h. According to Klinkaewnarong & Utara, heating above 800 °C converts the CaCO_3_ to CaO completely [82]. During the calcination, the carbon in the calcium carbonate will be converted to CO_2_ at temperatures between 700° to 900 °C [82]. The chemical reaction can be referred to Eq. (6).

Jamarun *et al.* studied the effect of precipitation and hydrothermal temperature on the synthesis of HAp from limestone [81, 83]. The research on the precipitation temperature was done at 60°, 70°, 80°, and 90 °C at pH 11 [81]. The CaO from the calcination of limestone was dissolved in 2 M HNO_3_ and 0.6 M ((NH_4_)_2_HPO_4_) and stirred for 1 h at different temperatures (60 °C, 70 °C, 80 °C, and 90 °C). The solution were left for 1 day until the precipitate is formed. The precipitate was dried and calcined at 800 °C for 2 h. The XRD results showed that tricalcium phosphate was the major phase present at 60 °C, 70 °C, and 80 °C, while HAp was formed at 90 °C. EDS analysis revealed that the Ca/P ratio at 90 °C was higher than that seen in stoichiometric HAp. This indicates that at 90 °C, the HAp phase was mixed with other CaP phases. The morphological analysis by SEM showed that the HAp was spherical in shape with sizes between 22.5-68.5 nm.

Jamarun *et al.* used a combination of calcination and hydrothermal techniques to synthesis HAp [83]. The calcined limestone was mixed with 2 M HNO_3_ and 0.6 M ((NH_4_)_2_HPO_4_), stirred for 30 min at 65 °C before hydrothermal treatment at 120 °C, 160 °C, and 200 °C for 24 h. The precipitate were then dried and calcined again at 800 °C for 5 h. XRD results showed that at 200 °C, HAp was formed and at lower hydrothermal temperatures, a mixed phase of HAp and CaP were produced. The Ca/P ratio of all samples were lower than 1.67 and the particles were spherical with sizes of 30–60 nm.

Klinkaewnarong & Utara used a combination of calcination and ultrasonic irradiation techniques [82]. This technique uses ultrasonic waves to induce synthesis of HAp. In this method, calcined limestone was mixed with 2.03 M H_3_PO_4_ and the pH was adjusted to pH 4 before proceeding with ultrasonic treatment. The frequency of the ultrasonic waves was 20 kHz at 25 °C and irradiation was done for 0, 1, 2, 3, and 4 h. The precipitate was washed using deionised water until the pH was 7 and then dried and calcined again at 300 °C for 3 h. All samples contained HAp and monetite or anhydrous dicalcium phosphate (DCPA, CaHPO_4_). The XRD peaks of monetite decreased as the sonication time was prolonged. Klinkaewnarong & Utara suggested that the ultrasonic irradiation affected the chemical reaction by altering the rate of formation and chemical equilibrium of HAp; thus ultrasonic irradiation method might produce more HAp monetite phase [82]. TEM analysis revealed that with increase in irradiation time, smaller sized, needle-like nanoparticles were produced. The optimal ultrasonic irradiation time was 3 h which produced needle-like and plate like morphology with average sizes of 7.4 nm width and 62.5 nm length. From the results, Klinkaewnarong & Utara conclude that ultrasonic waves induced HAp formation as a result of physical and chemical effects of cavitation and the combination of these effects decrease the interference from other chemical species such as Al, Si, Mg, and Fe and also accelerated the reaction of chemical species (CaO, H_3_PO_4_, CaHPO_4_ and H_2_O). According to Klinkaewnarong & Utara, the chemical reactions of the limestone to produce HAp are summarised as Eqs. (13), (14), and (15) [82].

(13) $${\text{CaCO}}_{3}\overset{1000\text{°C}\;}{\underset{\Delta }{\to }}\text{CaO}\;+\;{\text{CO}}_{2}$$

(14) $$\text{CaO}\;+{\;\text{H}}_{3}{\text{PO}}_{4}\overset{\text{0,}\;1\text{,}\;2\;\text{h}\;\;}{\underset{\text{Ultrasonic}\;\text{irradiation}}{\to }}\begin{array}{c} {\text{CaHPO}}_{4}\;+{\;\text{H}}_{2}\text{O}\\ \text{Monetite}\;\text{phase} \end{array}$$

(15) $$10\;{\text{CaHPO}}_{4}\;+\;{2\text{H}}_{2}\text{O}\overset{3\text{,}\;4\;\text{h}\;\;}{\underset{\text{Ultrasonic}\;\text{irradiation}}{\to }}\begin{array}{c} {{{\text{Ca}}_{10}\text{(}{\text{PO}}_{4}\text{)}}_{6}\text{(OH)}}_{2}\;+\;4{\;\text{H}}_{3}{\text{PO}}_{4}\\ \text{Hydroxyapatite}\;\text{phase} \end{array}$$

Table 7 list several combinations of methods used for the production of HAp from mineral sources. All methods used calcination as one of the approaches and thus all the extracted HAp showed high crystallinity. All of the methods were seen to not produce pure HAp with traces of other calcium phosphate phases seen similar to the case of extraction from shell and algae.

**Table 7:** Summary of the properties of HAp extracted from mineral sources.

Source	Method of extraction	Ca/P ratio	Crystalline Phases	Particle size	Shape	Reference (s)
Limestone	Calcination + precipitation	<1.67	HAp + CaO	22.5–68.5 nm	Spherical	[81]
Limestone	Calcination + hydrothermal	1.293 (160 °C) 1.296 (200 °C)	HAp + CaP (160 °C) HAp (200 °C)	32–46 nm (160 °C) 34–60 nm (200 °C)	Spherical	[83]
Limestone	Calcination + ultrasonic irradiation	-	HAp + CaHPO_4_	7.4 nm width and 62.5 nm length (3h)	Needle-like, plate-like	[82]

**Fig. 7:**
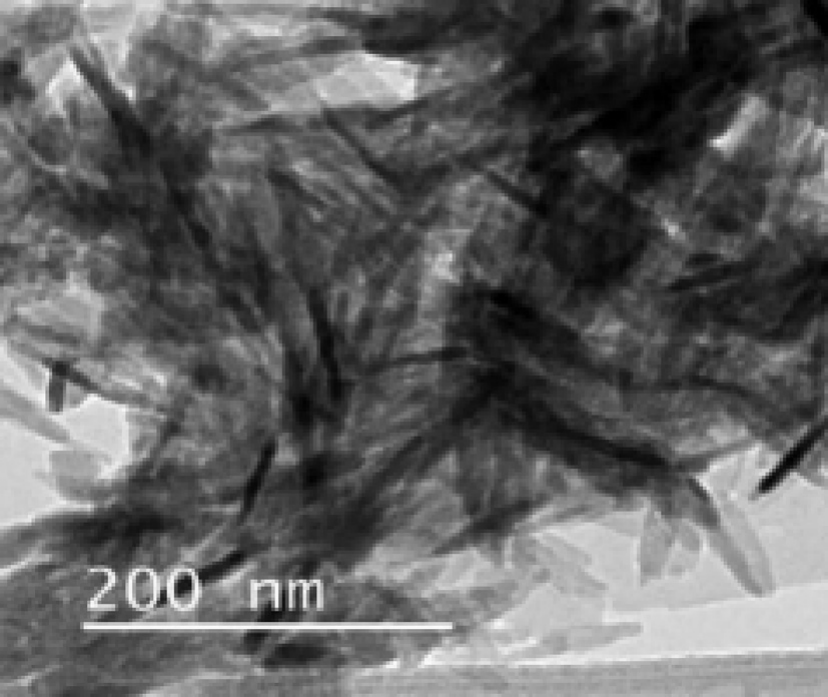
SEM micrograph of HAp extracted from limestone extracted using a combination of calcination and ultrasonic irradiation. (Adapted from [82], with permission from Elsevier).

The morphological analysis of the extracted HAp revealed spherical, needle-like, and plate-like shape with sizes <70 nm. Fig. 7 shows the SEM micrograph of HAp extracted from limestone using combination of calcination and hydrothermal methods.

The Ca/P ratios of the extracted HAp from limestone also are non-stoichiometric. The combinations of calcination and precipitation method produces HAp with Ca/P ratio greater than 1.67 while the use of calcination and hydrothermal method produces low Ca/P ratio (1.293 and 1.296). The greater Ca/P ratio of HAp in calcination and precipitation method was due to the presence of CaO in the extracted HAp. On the other hand, the lower Ca/P ratio using calcination and hydrothermal method may be due to the presence of other calcium phosphate phases with lower Ca/P ratio like β-TCP (1.5) or amorphous calcium phosphate (1.2–2.2).

### 2.3 Summary of hydroxyapatite extraction from all natural sources

#### 2.3.1 Properties of the hydroxyapatite

In summary, most natural sources have the potential to be a precursor for HAp production. The use of animal (mammalian and aquatic/marine), shell waste, plants, and minerals results in HAp of differing characteristics. Thus, Table 8 compares the properties of HAp extracted from different sources.

**Table 8:** Summary of the properties of HAp extracted from all different sources.

	Source				
Properties	Mammalian	Aquatic/marine	Shell	Plant/algae	Mineral
Morphology	Irregular, rod-like, flakes-like, needle-like, plate-like	Flat-plate, hexagonal, rod-like, irregular, nearly spherical, agglomerate, vary	Spherical, needle-like, rod like, globules, agglomerate polygonal	Flakes, cluster, rectangular, elongate	Spherical, needle-like, plate-like
Particle size	20–900 nm	5–1870 nm	5 nm-10.4 μm	50–500 nm	7.4–68.5 nm
Crystallinity	• High (calcination) • Low (chemical treatment)	• High (calcination) • Low (chemical treatment)	• High (combination method) • Low (chemical treatment	• High (calcination) • Low (chemical treatment	• High (combination method)
Ca/P ratio	1.5–2.131	1.60–2.01	1.425–1.8	1.53–1.72	1.293-<1.67
Presence of trace element	• Mg, Na, Zn, Sr, Ba [22] • Mg, Na [26] • Mg, Na, Sr, Fe, Al, Zn [27]	• Mg [47] • Mg, Sr,Na, K [45]	-	-	-

The morphological analysis shows that the frequent shape seen for HAp is rod-like or needle like. As mentioned previously, the shape of the HAp particles was not affected by the extraction method or the source.

Research is focussed on synthesising nanosize HAp owing to its superior properties and similarity in size to HAp in human bone (50–100 nm length and 1–10 nm diameter) [84]. Most methods use an additional milling step in order to achieve the nanosize of the HAp particle. However, HAp from fish scales does not require the milling process; however an alkaline hydrolysis method was used to enhance the dissolution of the scale and reduce its size. Thus, as listed in Table 8, most of the extracted HAp have nanosize range.

The crystallinity of the HAp was dependent on the method of extraction. The usage of thermal method increased the crystallinity while the chemical precipitation method resulted in low crystallinity HAp.

On the other hand, the results showed that the Ca/P ratio from all natural sources were non-stoichiometric. In the case of mammalian and aquatic/marine sources, the presence of trace element might increase the HAp ratio to values higher than those seen for stoichiometric HAp. For other sources like shells, algae, and minerals, the presence of other calcium species such as CaO may result in increasing Ca/P value. The variation in temperature during calcination can affect the Ca/P value owing to the formation of β-TCP. The β-TCP has a Ca/P ratio of 1.5 [84,85] and is present at high calcination temperatures. β-TCP are usually obtained >850 °C [31].

Lastly, it can be seen in Table 8 that the HAp extracted from the mammalian and aquatic/marine sources contains trace elements which are not seen in the case of other sources, and these could have excellent potential for enhancing the biological properties of natural HAp.

Thus, by comparing all the sources, researchers can choose the best methods for achieving the excellent properties of HAp in term of simplicity of extraction method, high yields, and low costs.

#### 2.3.2 Optimum processing parameter for extraction of HAp

Various extraction method of HAp has been studied in order to extract the HAp from natural. These extraction methods result in the various properties of the extracted HAp. Out of this various technique, the calcination method, alkaline hydrolysis method and also combination method have been frequently used during the extraction process.

In calcination method, it can be seen that the pure HAp have been produced at the temperature range from 700 °C to 1000 °C. Result from Tables 1 and 3 above showed that the calcination of mammalian bone, fish scale, and fish bone within temperature range from 700 °C to 1000 °C able to produce pure HAp. Table 9 below listed the calcination temperature used that successfully extract pure HAp from different sources. Instead of pure crystalline HAp, there also the traces of β-TCP formed result in the degradation of the calcium phosphate at high temperature. β-TCP are usually obtained at temperature 850 °C and above [31]. Although at high temperature the β-TCP could be formed, many research listed in Table 9 shows that at high temperature up to 900 and 1000 °C, the pure crystalline HAp still can be produced.

**Table 9:** Calcination temperature for extraction of pure HAp.

Calcination temperature, °C					
Mammalian source				Aquatic/marine source	
Bovine	Camel	Horse	Pig	Fish scale	Fish bone
750 [21]	700 [27]	700 [27]	1000 [30]	800 [45]	900 [50]
900 [18]	1000 [26]			1000 [52]	1000 [47]

Alkaline hydrolysis method also has been usually used for extraction HAp from the natural source. This method was able to extract pure HAp with low crystalline properties. Usually, the sodium hydroxide has been used to hydrolyse the organic matter in the sources and separated it from the HAp component. Various range of sodium hydroxide has been used during the extraction method. The research shows that 20–25% NaOH able to remove all the organic matter in the bovine bone and result in production of pure HAp. On the other research, they used low concentration of NaOH from 5% and the process was repeated with high NaOH (50%) concentration to remove excess organic matter. Research by Venkatesan *et al.* was used low concentration of NaOH which is 2M (8%) NaOH for the extraction HAp from fish bone. The process was repeated until all the organic matter was completely removed. All in all, the usage of alkaline heat treatment were able to produce pure HAp without worrying of the calcium phosphate phase transformation that usually occur during the calcination process. Table 10 presented some of the NaOH concentration used during the alkaline hydrolysis process.

**Table 10:** Sodium hydroxide concentration used for extraction of pure HAp.

NaOH concentration, weight %		
Mammalian source	Aquatic/marine source	
Bovine	Fish scale	Fish bone
25 % [20]	5 % and 50 % [49]	2M (8%) [50]
20 % [22]		

Apart from the calcination method and alkaline hydrolysis method, combination methods have been frequently used for the extraction of pure HAp. According to Sadat-Shojai *et al.* the used of combination were to improve the final properties of the product which is HAp. The use of combination may enhance the properties of the extracted HAp. For example the combination of alkaline hydrolysis and calcination method will increase the crystallinity of the HAp. In addition according to Sadat-Shojai *et al.* the used of combination calcination and rapid microwave irradiation increase the reaction kinetics and effectively reduce duration of the process and thus improved HAp properties such as reducing size, high purity, and narrower size distribution.

On the other hand, these combination methods were usually being used for production of HAp from the CaCO_3_ source such as from shell source, algae and also from limestone. During the process, calcination of the CaCO_3_ source were necessary before proceed with the other method that will transformed the calcium phosphate phase to HAp. After conversion of CaCO_3_ to CaO, usually the CaO will be mixed with additional phosphate source using other method such as chemical precipitation, sol gel or mechanochemical method in order to complete the conversion to HAp. Thus, several chemical transformation were involved during the process depend on the type of phosphate source that have been used. Thus, this combination method was very important in order to convert the CaCO_3_ source to pure HAp. Table 11 listed of combination method used for production of HAp.

**Table 11:** Combination methods used for extraction of HAp.

Source	Method	Reference(s)
Bovine bone	Hydrothermal + calcination	[23]
Pig bone	Alkaline hydrolysis + calcination	[32, 33]
Fish scale	Alkaline hydrolysis + calcination	[44]
Cockle shell	Calcination + sol gel	[60]
	Calcination + hydrothermal	[62]
Clam shell	Calcination + mechanochemical	[63]
Sea shell	Calcination + chemical precipitation	[61]
Egg shell	Calcination + chemical precipitation	[64]
Mussel shell	Calcination + rapid microwave irradiation	[66]
Limestone	Calcination + chemical precipitation	[81]
	Calcination + hydrothermal	[83]
	Calcination + ultrasonic irradiation	[82]

## 3 Conclusion

This review summarizes some recent methods that have been applied in synthesising HAp from natural sources such as mammalian, marine and aquatic, shell, plant and algae and mineral sources. In general, the HAp produced from different sources was distinct in terms of morphology and sizes. The characteristics of the obtained HAp were dependent on the method of extraction with thermal methods (calcination) producing highly-crystalline HAp compared to chemical precipitation. Some sources such as seashell and algae are rich in CaCO_3_ which is treated further to transform it into pure phase of HAp. Thus, various methods have been used either solely or in combination in order to produce high purity HAp with Ca/P ratio close to that of human bone minerals. Due to the increasing demand for HAp in biomedical applications, it is crucial to further understand the properties of the HAp such as phase purity and thermal stability especially when extracted from natural sources. In addition, the use of natural sources to extract HAp ensures sustainability since these natural sources can allow for nutrient recovery of waste materials in order to transform them into value added materials.

## Declarations

### Author contribution statement

All authors listed have significantly contributed to the development and the writing of this article.

### Funding statement

The authors gratefully acknowledge Universiti Tun Hussein Onn Malaysia and Ministry of Education Malaysia for the financial support provided for this research through Research Grant Scheme, TIER 1 Vot U880, GPPS Vot U978 and FRGS Vot K097.

### Competing interest statement

The authors declare no conflict of interest.

### Additional information

No additional information is available for this paper.
